# Impacts of Imperfect Channel State Information, Transceiver Hardware, and Self-Interference Cancellation on the Performance of Full-Duplex MIMO Relay System

**DOI:** 10.3390/s20061671

**Published:** 2020-03-17

**Authors:** Ba Cao Nguyen, Nguyen Nhu Thang, Xuan Nam Tran, Le The Dung

**Affiliations:** 1 Telecommunications University, Nha Trang 650000, Vietnam; nguyenbacao@tcu.edu.vn (B.C.N.); nguyennhuthang@tcu.edu.vn (N.N.T.); 2 Le Quy Don Technical University, Ha Noi 100000, Vietnam; namtx@mta.edu.vn; 3 Division of Computational Physics, Institute for Computational Science, Ton Duc Thang University, Ho Chi Minh City 700000, Vietnam; 4 Faculty of Electrical and Electronics Engineering, Ton Duc Thang University, Ho Chi Minh City 700000, Vietnam

**Keywords:** hardware impairments, full-duplex, multiple-input multiple-output, channel state information, outage probability, ergodic capacity

## Abstract

Imperfect channel state information (I-CSI) and imperfect transceiver hardware often happen in wireless communication systems due to the time-varying and random characteristics of both wireless channels and hardware components. The impacts of I-CSI and hardware impairments (HI) reduce not only the system performance but also the self-interference cancellation (SIC) capability of full-duplex (FD) devices. To investigate the system performance in realistic scenarios, in this paper, we consider the performance of an FD multiple-input multiple-output (MIMO) relay system under the effects of I-CSI, imperfect SIC (I-SIC), and imperfect transceiver hardware. We mathematically derive the exact closed-form expressions of the outage probability (OP) and ergodic capacity of the considered HI-FD-MIMO relay system over Rayleigh fading channels with the existence of I-CSI, I-SIC, and HI. Numerical results indicate that the performance in terms of OP and capacity reaches saturation faster, especially when the channel estimation error, the residual self-interference (RSI), and HI levels are remarkable. Therefore, various solutions for effectively reducing the channel estimation error, RSI, and HI levels in the HI-FD-MIMO relay system should be carried out to improve the system performance. All derived mathematical expressions are verified through Monte-Carlo simulations.

## 1. Introduction

Recently, full-duplex (FD) communication has greatly benefited future wireless networks due to its capability of doubling the spectral efficiency for wireless systems [1,2,3,4]. Consequently, FD devices are applied in various wireless communication systems such as cognitive radio, cooperative networks, device-to-device (D2D), multiple-input multiple-output (MIMO) communications, and relay networks [3,5,6]. Additionally, with the fast development of internet of things (IoT) and unmanned aerial vehicle (UAV) communication systems [7,8,9,10], FD can combine with many advanced techniques such as MIMO, nonorthogonal multiple access (NOMA) to enhance the performance of wireless systems. The combination of FD and MIMO techniques into wireless systems can significantly enhance the system capacity compared with traditional half-duplex (HD) or single-input single-output (SISO) systems [11,12,13]. However, using multiple radio frequency (RF) chains in MIMO systems causes difficulties in hardware deployment and signal processing, especially for small mobile users. Therefore, maximal ratio transmission (MRT) at the transmitter and maximal ratio combining (MRC) at the receiver were proposed to reduce the number of RF chains [14,15]. It is shown that applying MRT/MRC techniques not only improves the performance significantly but also provides full diversity order for MIMO systems. Thus, MRT/MRC techniques have been widely used in MIMO systems [16].

In the literature, employing FD technique at relays to enhance the reliability and coverage of FD-MIMO systems has been widely considered. Specifically, FD technique was utilized to improve the spectral efficiency of massive MIMO systems [17], the capacity and throughput of other wireless communication systems such as mmWave [18] and vehicle-to-vehicle (V2V) [19,20]. The authors of [21] analyzed the performance in terms of the outage probability (OP) and the ergodic capacity of an FD-MIMO relay system with amplify-and-forward (AF) protocol and under the presence of co-channel interference. From the exact closed-form expressions of OP and capacity, they demonstrated that the performance, especially the ergodic capacity, of FD-MIMO relay system with perfect channel state information (P-CSI) and ideal hardware is better compared with traditional HD-MIMO relay system. In [22], the authors calculated the upper and lower bounds of achievable data rate for FD-MIMO relay system with a decode-and-forward (DF) protocol under the impact of the limited dynamic range of the input circuitry. The results indicated that the attainable data rate was saturated because of the limited dynamic range. The authors of [23] determined the end-to-end achievable rate, where the hardware impairment (HI) is treated as distortion noises, to investigate the impact of HIs induced by low-cost hardware components on the performance of FD-MIMO relay system. Simulation results showed that the HI significantly reduces the attainable data rate of the FD-MIMO relay system. Besides, some recent reports about FD-MIMO relay systems also investigated the average error-probability performance in the case of imperfect self-interference cancellation (I-SIC) [24], the impact of HI on the spectral efficiency [25], and the achievable sum-rate [26].

It is evident that FD-MIMO relay systems now become promising candidates for future wireless networks such as 5G and beyond. It is because FD-MIMO relay systems not only have higher capacity but also lower feedback and transmission delay compared with traditional HD-MIMO relay systems. When applying precoding signals and advantaged detectors such as zero-forcing (ZF), minimum mean square error (MMSE), and maximum-likelihood (ML), the system performance of the FD-MIMO relay systems can be improved further [13,21,22,23,25]. On the other hand, using MRT/MRC techniques helps to decrease the complexities of hardware deployment and signal processing in various scenarios because MRT/MRC techniques are simple signal processing schemes and combine signals in such a way that the signal-to-noise ratio (SNR) at the output of receiver combiner is maximized. We observe that, although the impacts of the RSI caused by I-SIC and the HI due to the nonideal hardware were investigated, closed-form mathematical expressions of OP and capacity of the system with the I-SIC and HI were not derived. In addition, although there have been a lot of studies on the performance of FD-MIMO relay systems for both AF and DF protocols, they focused on the capacity or achievable rates of the system. Meanwhile, we need to increase the capacity of wireless systems with a low degradation on the performance of OP and bit/symbol error rate (BER/SER). Thus, we should consider OP or BER/SER and capacity at the same time when evaluating the systems because a wireless system with a high capacity but low OP or BER/SER performance cannot be used.

To consider realistic wireless systems and tackle the issues mentioned earlier in previous works on the FD-MIMO relay systems, in this paper, we investigate an FD-MIMO relay system with HI (namely, the HI-FD-MIMO relay system) with I-CSI and I-SIC. We evaluate the impacts of three imperfect factors—i.e., I-CSI, imperfect transceiver hardware, and I-SIC—on the OP and ergodic capacity of the considered HI-FD-MIMO relay system. So far, our paper is the first work that considers three imperfect factors—i.e., I-CSI, I-SIC, and HI—in the FD-MIMO relay system. From the mathematical expressions in our paper, we can easily obtain the OP and ergodic capacity of the FD-MIMO relay system with perfect CSI, perfect hardware, and perfect SIC, so that the performance of FD-MIMO relay systems in the case of three imperfect factors can be compared with that in the case of none, one, or two imperfect factors. It is also noted that, besides OP and capacity, BER is an important parameter for the system performance. However, due to the existence of three imperfect factors, it is too difficult to derive the upper-bound or lower-bound BER of the considered HI-FD-MIMO relay system. Therefore, in the paper, we focus on mathematically analyzing the OP and the ergodic capacity. The main contributions of the paper are shortened as follows:

We investigate an FD-MIMO relay system where imperfect CSI, imperfect transceiver hardware, and imperfect SIC coexist. Firstly, we obtain the received signals at relay and destination. Then, we derive the signal-to-interference-plus-noise-and-distortion ratio (SINDR) of the considered HI-FD-MIMO relay system with I-CSI, I-SIC, and HI.

We derive the exact closed-form expressions of the OP and ergodic capacity of the considered HI-FD-MIMO relay system with MRT/MRC techniques over Rayleigh fading channels. Based on these expressions, we can quickly obtain the expressions of the OP and ergodic capacity of the FD-MIMO relay system in the case of P-CSI, P-SIC, and ideal hardware. Monte-Carlo simulations are used to verify the exactness of our analysis.

We analyze the OP and ergodic capacity of the considered system. We also compare with the OP and ergodic capacity in the case of P-CSI, P-SIC, and ideal hardware to evaluate the sole effect of each imperfect factor and the combined effects of all imperfect factors. The results show that each imperfect factor has a strong influence on the OP and ergodic capacity of the considered system. When all three imperfect factors exist in the system, the OP performance is greatly reduced, and the ergodic capacity is very limited. Both OP and capacity reach the floor due to the existence of I-CSI, I-SIC, and HI. Therefore, various solutions need to be applied to the HI-FD-MIMO relay system to reduce the channel estimation error and the HI, and improve the SIC capability so that we can achieve better system performance.

The remainder of the paper is organized as follows. Section 2 presents the system and signal models where I-CSI, I-SIC, and HI are taken into account. Section 3 mathematically derives the closed-form expressions of the OP and ergodic capacity of the considered HI-FD-MIMO relay system. Section 4 provides numerical results and discussion. Finally, Section 5 concludes this paper.

## 2. System Model

**Figure 1:**
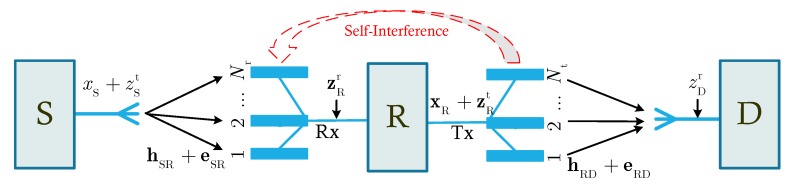
Block diagram of the considered HI-FD-MIMO relay system. HI—hardware impairment, FD—full-duplex, MIMO—multiple-input multiple-output.

The block diagram of the considered HI-FD-MIMO relay system is illustrated in Figure 1. The signals are transmitted from a single antenna device, i.e., source (S), to another single antenna device, i.e., destination (D), via the help of a multiple antenna device, i.e., relay (R). The FD transmission mode is exploited at the R to enhance the spectral efficiency of the system. However, due to the FD transmission, R experiences the self-interference (SI) from its transmission antennas to reception antennas. Figure 1 clearly indicates that the transmitted and received signals at the nonideal hardware devices are distorted by the HI. Specifically, the transmitted signal at S is $x_{S}+z_{S}^{t}$ for the HI system while it is only $x_{S}$ for the ideal hardware system. In other words, $x_{S}$ and $x_{S}+z_{S}^{t}$ are the intended and actual signals transmitted from S, respectively. It is similar for the receivers such as R and D in the system. In addition, R has $N_{r}$ reception antennas and $N_{t}$ transmission antennas. We should notice that R can use shared antennas for both transmitting and receiving signals. However, when using separate antennas for signal transmission and reception, R can do SIC better, especially for passive cancellation methods [27].

In the case of imperfect CSI, the channel from S to R or from R to D is expressed as

(1) $$\begin{array}{c} {\hat{h}}_{ij}=h_{ij}+e_{ij}, \end{array}$$

where ij∈{SR;RD}; $h_{ij}$ and $e_{ij}$ are the channel estimation vector and the channel estimation error vector, respectively. We assume that all elements of the channel estimation vector are subject to the Rayleigh distributions, while all elements of the channel estimation error vector are complex Gaussian random variables with zero means and variances of $\sigma_{e_{ij}}^{2}$ [28].

The received signal at R of the HI-MIMO-FD relay system is expressed as

(2) $$\begin{array}{c} y_{R}={\hat{h}}_{\mathrm{SR}}\left(x_{S}+z_{S}^{t}\right)+z_{R}^{r}+{\tilde{h}}_{\mathrm{RR}}\left(x_{R}+z_{R}^{t}\right)+n_{R}, \end{array}$$

where ${\hat{h}}_{\mathrm{SR}}={\left[{\hat{h}}_{S1}\;{\hat{h}}_{S2}\;\ldots \;{\hat{h}}_{SN_{r}}\right]}^{T}$ is the channel vector from the transmission antenna of S to $N_{r}$ reception antennas of R; $x_{S}$ is the intended signal which we want to transmit from S; $z_{S}^{t}$ is the HI noise caused by the transmitter S; $z_{R}^{r}$ is the HI noise vector caused by the receiver R; ${\tilde{h}}_{\mathrm{RR}}$ is the SI channel matrix from the transmission antennas to the reception antennas of R; $x_{R}={\left[x_{1}\;x_{2}\;\ldots \;x_{N_{t}}\right]}^{T}$ is the transmitted signal vector at $N_{t}$ transmission antennas of R; $z_{R}^{t}$ is the HI noise vector caused by the transmitter R; $n_{R}$ is the Gaussian noise vector at R and its elements have zero means and variances of $\sigma_{R}^{2}$, i.e., $n_{R}∼CN\left(0,\sigma_{R}^{2}\right)$.

As mentioned in the literature [29,30,31], the transceiver HI is caused by many factors, such as the in-phase/quadrature (I/Q) imbalance, high power amplifier (HPA) nonlinearities at the transmitter, and low noise amplifier (LNA) filters at the receiver [29,30,32]. Various compensation algorithms were applied to reduce the influence of the HI, but these methods could not remove HI completely. The residual HI still exists because of the time-varying and random hardware characteristics. Particularly, the HIs at the transmitters and the receivers after applying compensation algorithms can be modeled as Gaussian distributions [26,30,31], i.e., $z_{S}^{t}∼CN\left(0,{\left(k_{S}^{t}\right)}^{2}P_{S}\right)$, $z_{R}^{t}∼CN\left(0,{\left(k_{R}^{t}\right)}^{2}\frac{P_{R}}{N_{t}}\right)$, and $z_{R}^{r}∼CN(0,∥{\hat{h}}_{\mathrm{SR}}{∥}^{2}{\left(k_{R}^{r}\right)}^{2}P_{S})$, where, $k_{S}^{t}$, $k_{R}^{t}$, and $k_{R}^{r}$ respectively indicate the HI levels at the transmitters S and R and the receiver R; $P_{S}$ and $\frac{P_{R}}{N_{t}}$ are respectively the average transmission power per one antenna of S and R.

On the other hand, the full power of the SI due to FD transmission mode is ${\tilde{h}}_{\mathrm{RR}}\left(x_{R}+z_{R}^{t}\right)$, as given in (2). To detect the received signals transmitted from S successfully, R must apply all SIC techniques such as passive suppression and active cancellation [27,33]. Thanks to the usage of separate antennas for transmitting and receiving signals, R can use isolation, directional isolation, and absorptive shielding as passive suppression methods to reduce the SI power. Then, R can actively subtract the SI by estimating the SI channel. However, due to imperfect SI channel estimation and imperfect hardware, R cannot completely remove the SI from the received signals. Thus, the residual SI (RSI) will interfere with the intended signals. In addition, the RSI after all SIC techniques (denoted by $I_{R}$) follows Gaussian distribution, i.e., $I_{R}∼CN\left(0,\gamma_{\mathrm{RSI}}\right)$, where $\gamma_{\mathrm{RSI}}=l^{2}P_{R}$ with *l* denotes the SIC capability of the FDR device [5,33,34,35,36].

After SIC, the received signal at R becomes

(3) $$\begin{array}{c} y_{R}={\hat{h}}_{\mathrm{SR}}\left(x_{S}+z_{S}^{t}\right)+z_{R}^{r}+I_{R}+n_{R}. \end{array}$$

Using (1), we rewrite (3) as

(4) $$\begin{array}{cc} y_{R} & ={\hat{h}}_{\mathrm{SR}}x_{S}+{\hat{h}}_{\mathrm{SR}}z_{S}^{t}+z_{R}^{r}+I_{R}+n_{R}\\ \; & =h_{\mathrm{SR}}x_{S}+{\hat{h}}_{\mathrm{SR}}z_{S}^{t}+z_{R}^{r}+e_{\mathrm{SR}}x_{S}+I_{R}+n_{R}. \end{array}$$

Since the DF protocol is used, R decodes the received signals before forwarding them to D. Hence, the received signal at D in the case of I-CSI and HI is given by

(5) $$\begin{array}{cc} y_{D} & ={\hat{h}}_{\mathrm{RD}}\left(x_{R}+z_{R}^{t}\right)+z_{D}^{r}+n_{D}\\ \; & =h_{\mathrm{RD}}x_{R}+{\hat{h}}_{\mathrm{RD}}z_{R}^{t}+z_{D}^{r}+e_{\mathrm{RD}}x_{R}+n_{D}, \end{array}$$

where ${\hat{h}}_{\mathrm{RD}}=\left[{\hat{h}}_{1D}\;h_{2D}\;\ldots \;h_{N_{t}D}\right]$ is the channel vector from $N_{t}$ transmission antennas of R to the reception antenna of D; $z_{D}^{r}∼CN(0,∥{\hat{h}}_{\mathrm{RD}}{∥}^{2}{\left(k_{D}^{r}\right)}^{2}\frac{P_{R}}{N_{t}})$ is the HI at D, where $k_{D}^{r}$ is the HI level; $n_{D}∼CN\left(0,\sigma_{D}^{2}\right)$ is the Gaussian noise at D.

From the received signals at R and D in (4) and (5), we obtain the instantaneous signal-to-interference-plus-noise-and-distortion ratio (SINDR) at R and D (denoted by $\gamma_{R}$ and $\gamma_{D}$, respectively) as

(6) $$\begin{array}{cc} \gamma_{R} & =\frac{∥h_{\mathrm{SR}}{∥}^{2}P_{S}}{∥{\hat{h}}_{\mathrm{SR}}{∥}^{2}{\left(k_{S}^{t}\right)}^{2}P_{S}+{∥{\hat{h}}_{\mathrm{SR}}∥}^{2}{\left(k_{R}^{r}\right)}^{2}P_{S}+\sigma_{e_{\mathrm{SR}}}^{2}P_{S}+\gamma_{\mathrm{RSI}}+\sigma_{R}^{2}}\\ \; & =\frac{∥h_{\mathrm{SR}}{∥}^{2}P_{S}}{∥{\hat{h}}_{\mathrm{SR}}{∥}^{2}k_{S}^{2}P_{S}+\sigma_{e_{\mathrm{SR}}}^{2}P_{S}+\gamma_{\mathrm{RSI}}+\sigma_{R}^{2}}\\ \; & =\frac{∥h_{\mathrm{SR}}{∥}^{2}P_{S}}{∥h_{\mathrm{SR}}{∥}^{2}k_{S}^{2}P_{S}+\sigma_{e_{\mathrm{SR}}}^{2}P_{S}\left(1+k_{S}^{2}\right)+\gamma_{\mathrm{RSI}}+\sigma_{R}^{2}}, \end{array}$$

(7) $$\begin{array}{cc} \gamma_{D} & =\frac{∥h_{\mathrm{RD}}{∥}^{2}\frac{P_{R}}{N_{t}}}{∥{\hat{h}}_{\mathrm{RD}}{∥}^{2}{\left(k_{R}^{t}\right)}^{2}\frac{P_{R}}{N_{t}}+{∥{\hat{h}}_{\mathrm{RD}}∥}^{2}{\left(k_{D}^{r}\right)}^{2}\frac{P_{R}}{N_{t}}+\sigma_{e_{\mathrm{RD}}}^{2}P_{R}+\sigma_{D}^{2}}\\ \; & =\frac{∥h_{\mathrm{RD}}{∥}^{2}\frac{P_{R}}{N_{t}}}{∥{\hat{h}}_{\mathrm{RD}}{∥}^{2}k_{R}^{2}\frac{P_{R}}{N_{t}}+\sigma_{e_{\mathrm{RD}}}^{2}P_{R}+\sigma_{D}^{2}}\\ \; & =\frac{∥h_{\mathrm{RD}}{∥}^{2}P_{R}}{∥h_{\mathrm{RD}}{∥}^{2}k_{R}^{2}P_{R}+N_{t}\sigma_{e_{\mathrm{RD}}}^{2}P_{R}\left(1+k_{R}^{2}\right)+N_{t}\sigma_{D}^{2}}, \end{array}$$

where $k_{S}^{2}={\left(k_{S}^{t}\right)}^{2}+{\left(k_{R}^{r}\right)}^{2}$ is the aggregated HI level from both the transmitter side of S ($k_{S}^{t}$) and the receiver side of R ($k_{R}^{r}$); $k_{R}^{2}={\left(k_{R}^{t}\right)}^{2}+{\left(k_{D}^{r}\right)}^{2}$ is the aggregated HI level from both the transmitter side of R ($k_{R}^{t}$) and the receiver side of D ($k_{D}^{r}$).

## 3. Performance Analysis

### 3.1. Outage Probability Analysis

The OP is a criterion that is often used to evaluate the performance of wireless systems. It is defined as the probability that the instantaneous data transmission rate of the considered system falls below a certain data transmission rate [37]. Mathematically, the OP (denoted by $P_{\mathrm{out}}$) is calculated as

(8) $$\begin{array}{c} P_{\mathrm{out}}=\mathrm{Pr}\left\{R<R_{0}\right\}, \end{array}$$

where $R=\log_{2}\left(1+\gamma_{e2e}\right)$ is the instantaneous data transmission rate, $\gamma_{e2e}$ is the end-to-end SINDR of the HI-FD-MIMO relay system, and $R_{0}$ is the specified data transmission rate.

Since the DF protocol is applied at the FD relay, $\gamma_{e2e}$ is determined as

(9) $$\begin{array}{c} \gamma_{e2e}=\min\left\{\gamma_{R},\gamma_{D}\right\}, \end{array}$$

where $\gamma_{R}$ and $\gamma_{D}$ are given in (6) and (7), respectively.

Applying (9), we can rewrite (8) as

(10) $$\begin{array}{cc} P_{\mathrm{out}} & =\mathrm{Pr}\left\{\log_{2}\left(1+\gamma_{e2e}\right)<R_{0}\right\}=\mathrm{Pr}\left\{\gamma_{e2e}<2^{R_{0}}-1\right\}\\ \; & =\mathrm{Pr}\left\{\min\left\{\gamma_{R},\gamma_{D}\right\}<2^{R_{0}}-1\right\}=\mathrm{Pr}\left\{\min\left\{\gamma_{R},\gamma_{D}\right\}<\gamma_{\mathrm{th}}\right\}\\ \; & =\mathrm{Pr}\{\left(\gamma_{R}<\gamma_{\mathrm{th}}\right)\cup \left(\gamma_{D}<\gamma_{\mathrm{th}}\right)\}, \end{array}$$

where $\gamma_{\mathrm{th}}=2^{R_{0}}-1$ is the SINDR threshold.

Using the probability of two independent events, i.e., $\mathrm{Pr}\{\gamma_{R}<\gamma_{\mathrm{th}}\}$ and $\mathrm{Pr}\{\gamma_{D}<\gamma_{\mathrm{th}}\}$ [38], we have

(11) $$\begin{array}{c} P_{\mathrm{out}}=\mathrm{Pr}\left\{\gamma_{R}<\gamma_{\mathrm{th}}\right\}+\mathrm{Pr}\left\{\gamma_{D}<\gamma_{\mathrm{th}}\right\}-\mathrm{Pr}\left\{\gamma_{R}<\gamma_{\mathrm{th}}\right\}\mathrm{Pr}\left\{\gamma_{D}<\gamma_{\mathrm{th}}\right\}. \end{array}$$

Based on (11), we obtain the OP of the considered HI-FD-MIMO relay system as follows in Theorem 1.

**Theorem** **1.** *Under the impact of the channel estimation error, HI, and RSI, the OP expression of the considered HI-FD-MIMO relay system is given by*

(12) $$\begin{array}{c} P_{\mathrm{out}}=\left\{\begin{array}{cc} 1-\exp\left(-\frac{A\gamma_{\mathrm{th}}}{1-k_{S}^{2}\gamma_{\mathrm{th}}}-\frac{B\gamma_{\mathrm{th}}}{1-k_{R}^{2}\gamma_{\mathrm{th}}}\right)\sum_{i=0}^{N_{r}-1}\sum_{j=0}^{N_{t}-1}\frac{1}{i!j!}{\left(\frac{A\gamma_{\mathrm{th}}}{1-k_{S}^{2}\gamma_{\mathrm{th}}}\right)}^{i}{\left(\frac{B\gamma_{\mathrm{th}}}{1-k_{R}^{2}\gamma_{\mathrm{th}}}\right)}^{j}, & \gamma_{\mathrm{th}}<\min\left(\frac{1}{k_{R}^{2}},\frac{1}{k_{S}^{2}}\right)\\ 1, & \gamma_{\mathrm{th}}\geq \min\left(\frac{1}{k_{R}^{2}},\frac{1}{k_{S}^{2}}\right), \end{array}\right. \end{array}$$

where $A=\frac{\sigma_{e_{\mathrm{SR}}}^{2}P_{S}\left(1+k_{S}^{2}\right)+\gamma_{\mathrm{RSI}}+\sigma_{R}^{2}}{\Omega_{1}P_{S}}$; $B=\frac{N_{t}\left[\sigma_{e_{\mathrm{RD}}}^{2}P_{R}\left(1+k_{R}^{2}\right)+\sigma_{D}^{2}\right]}{\Omega_{2}P_{R}}$; and $\Omega_{1}$ and $\Omega_{2}$ are the average channel gains of the communication links from S to R and from R to D, respectively.

**Proof** **of Theorem 1.** As can be seen from (11), we need to obtain two probabilities, i.e., $\mathrm{Pr}\{\gamma_{R}<\gamma_{\mathrm{th}}\}$ and $\mathrm{Pr}\{\gamma_{D}<\gamma_{\mathrm{th}}\}$, to derive the OP of the considered HI-FD-MIMO relay system. Since the MRC technique is applied at R, the first probability $\mathrm{Pr}\{\gamma_{R}<\gamma_{\mathrm{th}}\}$ is calculated as

(13) $$\begin{array}{c} \mathrm{Pr}\left\{\gamma_{R}<\gamma_{\mathrm{th}}\right\}=\mathrm{Pr}\left\{\frac{∥h_{\mathrm{SR}}{∥}^{2}P_{S}}{∥h_{\mathrm{SR}}{∥}^{2}k_{S}^{2}P_{S}+\sigma_{e_{\mathrm{SR}}}^{2}P_{S}\left(1+k_{S}^{2}\right)+\gamma_{\mathrm{RSI}}+\sigma_{R}^{2}}<\gamma_{\mathrm{th}}\right\}, \end{array}$$

which is equivalent to

(14) $$\begin{array}{c} \mathrm{Pr}\left\{\gamma_{R}<\gamma_{\mathrm{th}}\right\}=\mathrm{Pr}\left\{∥h_{\mathrm{SR}}{∥}^{2}P_{S}\left(1-k_{S}^{2}\gamma_{\mathrm{th}}\right)<\gamma_{\mathrm{th}}\left[\sigma_{e_{\mathrm{SR}}}^{2}P_{S}\left(1+k_{S}^{2}\right)+\gamma_{\mathrm{RSI}}+\sigma_{R}^{2}\right]\right\}. \end{array}$$

To solve the probability in (14), we need to consider two cases: $1-k_{S}^{2}\gamma_{\mathrm{th}}\leq 0$ and $1-k_{S}^{2}\gamma_{\mathrm{th}}>0$.

In the case of $1-k_{S}^{2}\gamma_{\mathrm{th}}\leq 0$ or $\gamma_{\mathrm{th}}\geq \frac{1}{k_{S}^{2}}$, we see that $∥h_{\mathrm{SR}}{∥}^{2}P_{S}\left(1-k_{S}^{2}\gamma_{\mathrm{th}}\right)\leq 0$ and $\gamma_{\mathrm{th}}\left[\sigma_{e_{\mathrm{SR}}}^{2}P_{S}\left(1+k_{S}^{2}\right)+\gamma_{\mathrm{RSI}}+\sigma_{R}^{2}\right]>0$. Thus, the probability in (14) always occurs. In other words, $\mathrm{Pr}\{\gamma_{R}<\gamma_{\mathrm{th}}\}=1$ when $\gamma_{\mathrm{th}}\geq \frac{1}{k_{S}^{2}}$.

In the case of $1-k_{S}^{2}\gamma_{\mathrm{th}}>0$ or $\gamma_{\mathrm{th}}<\frac{1}{k_{S}^{2}}$, (14) can be rewritten as

(15) $$\begin{array}{c} \mathrm{Pr}\left\{\gamma_{R}<\gamma_{\mathrm{th}}\right\}=\mathrm{Pr}\left\{∥h_{\mathrm{SR}}{∥}^{2}<\frac{\gamma_{\mathrm{th}}\left[\sigma_{e_{\mathrm{SR}}}^{2}P_{S}\left(1+k_{S}^{2}\right)+\gamma_{\mathrm{RSI}}+\sigma_{R}^{2}\right]}{P_{S}\left(1-k_{S}^{2}\gamma_{\mathrm{th}}\right)}\right\}. \end{array}$$

Since $∥h_{\mathrm{SR}}{∥}^{2}=|h_{S1}{|}^{2}+|h_{S2}{|}^{2}+\ldots +{\left|h_{SN_{r}}\right|}^{2}$, the probability density function (PDF, f(.)) and the cumulative distribution function (CDF, F(.)) of $∥h_{\mathrm{SR}}{∥}^{2}$ are respectively given as follows [2,39]:

(16) $$\begin{array}{c} F_{∥h_{\mathrm{SR}}{∥}^{2}}\left(x\right)=1-\exp\left(-\frac{x}{\Omega_{1}}\right)\sum_{i=0}^{N_{r}-1}\frac{1}{i!}{\left(\frac{x}{\Omega_{1}}\right)}^{i},\;x\geq 0, \end{array}$$

(17) $$\begin{array}{c} f_{∥h_{\mathrm{SR}}{∥}^{2}}\left(x\right)=\frac{x^{N_{r}-1}}{\Omega_{1}^{N_{r}}\Gamma \left(N_{r}\right)}\exp\left(-\frac{x}{\Omega_{1}}\right),\;x\geq 0, \end{array}$$

where $\Omega_{1}=E\{|h_{S1}{|}^{2}\}=E\{|h_{S2}{|}^{2}\}=\cdots =E\{|h_{SN_{r}}{|}^{2}\}$ is the average channel gain of the communication links from S to R, with E denotes the expectation operator; Γ(.) is the gamma function [40].

From the CDF of $∥h_{\mathrm{SR}}{∥}^{2}$ in (16), we can transform (15) as

(18) $$\begin{array}{cc} \mathrm{Pr}\{\gamma_{R}<\gamma_{\mathrm{th}}\}= & F_{∥h_{\mathrm{SR}}{∥}^{2}}\left(\frac{\gamma_{\mathrm{th}}\left[\sigma_{e_{\mathrm{SR}}}^{2}P_{S}\left(1+k_{S}^{2}\right)+\gamma_{\mathrm{RSI}}+\sigma_{R}^{2}\right]}{P_{S}\left(1-k_{S}^{2}\gamma_{\mathrm{th}}\right)}\right)\\ = & 1-\exp\left(-\frac{\gamma_{\mathrm{th}}\left[\sigma_{e_{\mathrm{SR}}}^{2}P_{S}\left(1+k_{S}^{2}\right)+\gamma_{\mathrm{RSI}}+\sigma_{R}^{2}\right]}{\Omega_{1}P_{S}\left(1-k_{S}^{2}\gamma_{\mathrm{th}}\right)}\right)\\ \; & \times \sum_{i=0}^{N_{r}-1}\frac{1}{i!}{\left(\frac{\gamma_{\mathrm{th}}\left[\sigma_{e_{\mathrm{SR}}}^{2}P_{S}\left(1+k_{S}^{2}\right)+\gamma_{\mathrm{RSI}}+\sigma_{R}^{2}\right]}{\Omega_{1}P_{S}\left(1-k_{S}^{2}\gamma_{\mathrm{th}}\right)}\right)}^{i}\\ = & 1-\exp\left(-\frac{A\gamma_{\mathrm{th}}}{1-k_{S}^{2}\gamma_{\mathrm{th}}}\right)\sum_{i=0}^{N_{r}-1}\frac{1}{i!}{\left(\frac{A\gamma_{\mathrm{th}}}{1-k_{S}^{2}\gamma_{\mathrm{th}}}\right)}^{i}, \end{array}$$

where *A* is defined as in Theorem 1.

Combining two previous cases, we obtain the probability $\mathrm{Pr}\{\gamma_{R}<\gamma_{\mathrm{th}}\}$ as

(19) $$\begin{array}{c} \mathrm{Pr}\left\{\gamma_{R}<\gamma_{\mathrm{th}}\right\}=\left\{\begin{array}{cc} 1-\exp\left(-\frac{A\gamma_{\mathrm{th}}}{1-k_{S}^{2}\gamma_{\mathrm{th}}}\right)\sum_{i=0}^{N_{r}-1}\frac{1}{i!}{\left(\frac{A\gamma_{\mathrm{th}}}{1-k_{S}^{2}\gamma_{\mathrm{th}}}\right)}^{i}, & \gamma_{\mathrm{th}}<\frac{1}{k_{S}^{2}},\\ 1, & \gamma_{\mathrm{th}}\geq \frac{1}{k_{S}^{2}}. \end{array}\right. \end{array}$$

Similarly, the probability $\mathrm{Pr}\{\gamma_{D}<\gamma_{\mathrm{th}}\}$ can be calculated as

(20) $$\begin{array}{c} \mathrm{Pr}\left\{\gamma_{D}<\gamma_{\mathrm{th}}\right\}=\left\{\begin{array}{cc} 1-\exp\left(-\frac{B\gamma_{\mathrm{th}}}{1-k_{R}^{2}\gamma_{\mathrm{th}}}\right)\sum_{j=0}^{N_{t}-1}\frac{1}{j!}{\left(\frac{B\gamma_{\mathrm{th}}}{1-k_{R}^{2}\gamma_{\mathrm{th}}}\right)}^{j}, & \gamma_{\mathrm{th}}<\frac{1}{k_{R}^{2}},\\ 1,\gamma_{\mathrm{th}}\geq \frac{1}{k_{R}^{2}}. \end{array}\right. \end{array}$$

Replacing (19) and (20) into (11), the OP of the considered HI-FD-MIMO relay system is derived as (12) in Theorem 1. The proof is complete. □

### 3.2. Ergodic Capacity Analysis

The ergodic capacity of the considered HI-FD-MIMO relay system is calculated as [41]

(21) $$\begin{array}{c} C=E\left\{\log_{2}\left(1+\gamma_{e2e}\right)\right\}=\int_{0}^{\infty }\log_{2}\left(1+\gamma_{e2e}\right)f_{\gamma_{e2e}}\left(x\right)dx, \end{array}$$

where $\gamma_{e2e}$ and $f_{\gamma_{e2e}}\left(x\right)$ are respectively the end-to-end SINDR and its PDF.

Based on (21), the ergodic capacity of the HI-FD-MIMO relay system can be computed by

(22) $$\begin{array}{c} C=\frac{1}{\ln2}\int_{0}^{\infty }\frac{1-F_{\gamma_{e2e}}\left(x\right)}{1+x}dx, \end{array}$$

where $F_{\gamma_{e2e}}\left(x\right)$ is the CDF of $\gamma_{e2e}$.

From (22), the ergodic capacity of the considered HI-FD-MIMO relay system is derived in the following Theorem 2.

**Theorem** **2.** *The ergodic capacity of the considered HI-FD-MIMO relay system with I-CSI, I-SIC, and HI is expressed as*

(23) $$\begin{array}{c} C=\frac{\pi \Delta }{2M\ln2}\sum_{m=1}^{M}\sum_{i=0}^{N_{r}-1}\sum_{j=0}^{N_{t}-1}\frac{\sqrt{1-\phi_{m}^{2}}}{i!j!(1+\lambda )}\exp\left(-\frac{A\lambda }{1-k_{S}^{2}\lambda }-\frac{B\lambda }{1-k_{R}^{2}\lambda }\right){\left(\frac{A\lambda }{1-k_{S}^{2}\lambda }\right)}^{i}{\left(\frac{B\lambda }{1-k_{R}^{2}\lambda }\right)}^{j}, \end{array}$$

where M is the complexity-accuracy trade-off parameter for calculating the capacity; $\phi_{m}=\cos\left(\frac{(2m-1)\pi }{2M}\right)$; and $\lambda =\frac{\Delta }{2}\left(1+\phi_{m}\right)$.

**Proof** **of Theorem 2.** First, we calculate $F_{\gamma_{e2e}}\left(x\right)$ to replace it into (22). From the definition of $F_{\gamma_{e2e}}\left(x\right)$, we have

(24) $$\begin{array}{c} F_{\gamma_{e2e}}\left(x\right)=\mathrm{Pr}\left\{\gamma_{e2e}<x\right\}. \end{array}$$

We see that (24) is similar to the first line of (10). Therefore, we can obtain $F_{\gamma_{e2e}}\left(x\right)$ by using similar ways that were used to derive the OP. Finally, we obtain $F_{\gamma_{e2e}}\left(x\right)$ as

(25) $$\begin{array}{c} F_{\gamma_{e2e}}\left(x\right)=\left\{\begin{array}{cc} 1-\exp\left(-\frac{Ax}{1-k_{S}^{2}x}-\frac{Bx}{1-k_{R}^{2}x}\right)\sum_{i=0}^{N_{r}-1}\sum_{j=0}^{N_{t}-1}\frac{1}{i!j!}{\left(\frac{Ax}{1-k_{S}^{2}x}\right)}^{i}{\left(\frac{Bx}{1-k_{R}^{2}x}\right)}^{j}, & x<\Delta ,\\ 1, & x\geq \Delta , \end{array}\right. \end{array}$$

where $\Delta =\min\left(\frac{1}{k_{S}^{2}},\frac{1}{k_{R}^{2}}\right)$.

Substituting $F_{\gamma_{e2e}}\left(x\right)$ in (25) into (22), we have

(26) $$\begin{array}{c} C=\frac{1}{\ln2}\int_{0}^{\Delta }\frac{1}{1+x}\exp\left(-\frac{Ax}{1-k_{S}^{2}x}-\frac{Bx}{1-k_{R}^{2}x}\right)\sum_{i=0}^{N_{r}-1}\sum_{j=0}^{N_{t}-1}\frac{1}{i!j!}{\left(\frac{Ax}{1-k_{S}^{2}x}\right)}^{i}{\left(\frac{Bx}{1-k_{R}^{2}x}\right)}^{j}dx. \end{array}$$

Applying the Gaussian–Chebyshev quadrature method [42] Equation (25.4.30), the integral in (26) can be solved. Then, we obtain the ergodic capacity as (23) in Theorem 2. The proof is complete. □

## 4. Numerical Results and Discussions

In this section, the impacts of I-CSI, I-SIC, and HI on the OP and capacity of the considered HI-FD-MIMO relay system are investigated through the analytical expressions in Theorems 1 and 2. In all evaluating scenarios, the parameters are set as follows: the average transmission powers are $P_{S}=P_{R}=P$; the average channel gains are $\Omega_{1}=\Omega_{2}=1$; the Gaussian noises at the receivers are $\sigma_{R}^{2}=\sigma_{D}^{2}=\sigma^{2}=1$; the channel estimation errors are $\sigma_{e_{\mathrm{SR}}}^{2}=\sigma_{e_{\mathrm{RD}}}^{2}=\sigma_{e}^{2}$; the HI levels at the transceivers are $k_{S}^{t}=k_{R}^{r}=k_{R}^{t}=k_{D}^{r}=k$; the number of reception and transmission antennas of R are $N_{r}=N_{t}=3$; and the predata transmission rate is $R_{0}=4$ bit/s/Hz. The average SNR is defined as $\mathrm{SNR}=P/\sigma^{2}$.

**Figure 2:**
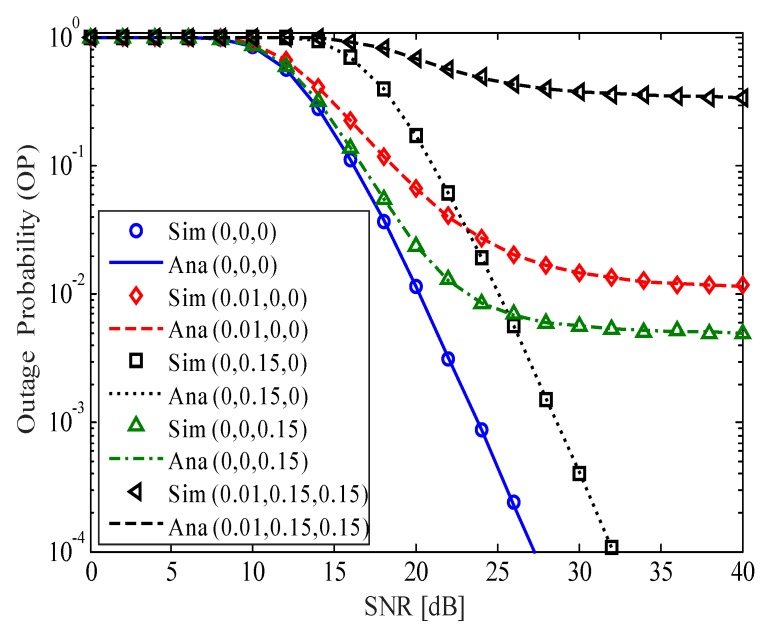
The outage probability (OP) of the considered HI-FD-MIMO relay system versus the average SNR for different values of $\sigma_{e}^{2}$, *k*, and *l*.

Figure 2 shows the OP of the considered HI-FD-MIMO relay system versus the average SNR for different values of $\sigma_{e}^{2}$, *k*, and *l*. The expression (12) is used to obtain the analysis curves and is verified by Monte-Carlo simulation results. In Figure 2, the triad (.,.,.) denotes ($\sigma_{e}^{2},k,l$). For example, (0,0.15,0) means $\sigma_{e}^{2}=0$, k=0.15, and l=0. We can see from Figure 2 that, compared with the perfect case (i.e., (0,0,0)), the impacts of I-CSI, I-SIC, and HI are very strong. Among the considered parameters, the impact of I-CSI on the OP of the considered system is the strongest. In the case of (0.01,0,0), the OP goes to the error floor faster at SNR=35dB with $\mathrm{OP}\approx 10^{-2}$. In the case of (0,0,0.15), the OP also reaches the error floor at SNR=35dB. However, the value of $\mathrm{OP}=8\times 10^{-3}$ is lower than that in the previous case. In the case of (0,0.15,0), the OP of the considered system has the same pattern with the perfect case. Although 5-dB-higher SNR is needed in comparison with the case of (0,0,0) at $\mathrm{OP}=10^{-4}$, the OP avoids reaching the error floor in the considered SNR range. When the three factors are imperfect (the case of (0.01,0.15,0.15)), the OP only reaches the outage floor of $4\times 10^{-1}$. Therefore, all solutions must be applied for the considered system to reduce the channel estimation error, HI, and RSI levels.

**Figure 3:**
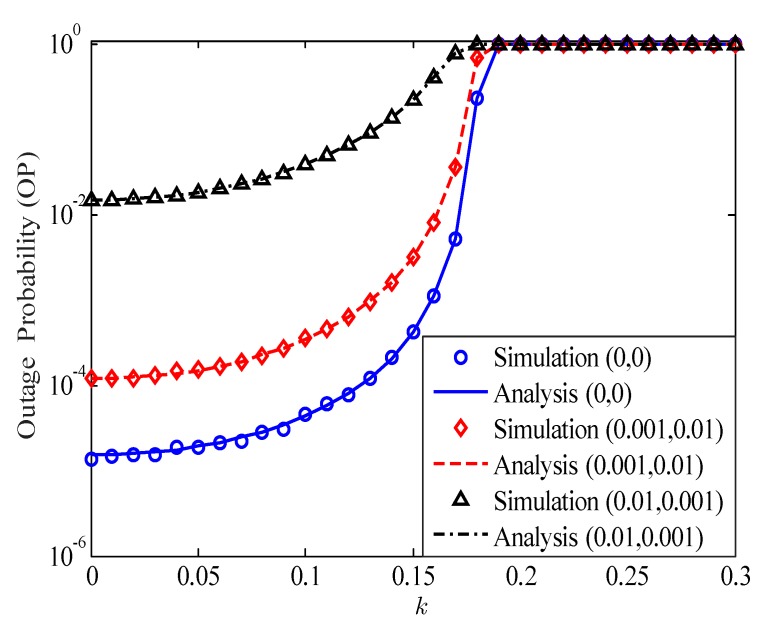
The impact of HI level on the OP of the considered HI-FD-MIMO relay system; SNR=30dB.

Figure 3 depicts the impact of HI level *k* on the OP of the considered HI-FD-MIMO relay system for different values of $\sigma_{e}^{2}$ and *l*. The dyad (.,.) in Figure 3 denotes $\sigma_{e}^{2}$ and *l*, e.g., (0.001,0.01) means $\sigma_{e}^{2}=0.001$ and l=0.01. It should be noted that, when k=0, we obtain the OP of the ideal hardware system. We can see from Figure 3 that, in the case of k≤0.05, the impact of the HI on the OP is very small and can be neglected. However, when k>0.05, the impact of the HI becomes stronger. Especially in the case of k=0.15, the OP performance is reduced approximately ten times compared with the case of k=0. With a higher value of *k*, e.g., k≥0.2, the OPs of the considered system go to 1 even with P-CSI and P-SIC (the case of (0,0)). Therefore, we need to apply all various solutions in both analog and digital signal processing to compensate for the hardware impairments for the FD-MIMO relay system.

**Figure 4:**
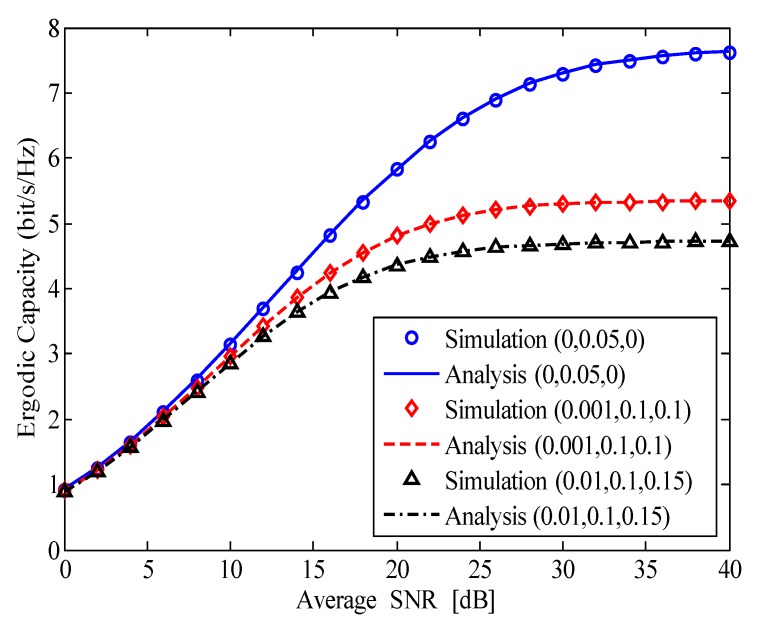
The ergodic capacity of the HI-FD-MIMO relay system versus the average SNR for different values of $\sigma_{e}^{2}$, *k*, and *l*.

Figure 4 illustrates the ergodic capacity of the considered HI-FD-MIMO relay system versus the average SNR for different values of $\sigma_{e}^{2}$, *k*, and *l*. Herein, the analysis curves are plotted by using (23) in Theorem 2. The meaning of the triad in Figure 4 is similar to that in Figure 2. In the first case of (0,0.05,0)—meaning $\sigma_{e}^{2}=0$, k=0.05, and l=0—the ergodic capacity of the considered system is significantly high. This is because, in this case, the impact of the HI can be neglected (refer to Figure 3); thus, the ergodic capacity is nearly ideal (i.e., the capacity is double compared with the traditional HD-MIMO relay system). In the case of $\sigma_{e}^{2}>0$ and l>0, e.g., (0.001,0.1,0.1) and (0.01,0.1,0.15), the ergodic capacity is reduced greatly compared with the first case. Particularly, at SNR=40dB, the first case can reach 7.7 bit/s/Hz while the second case of (0.001,0.1,0.1) and the third case of (0.01,0.1,0.15) only reach 5.3 and 4.8 bit/s/Hz, respectively. Thus, more efforts must be made to enhance the SIC capability and reduce the channel estimation error and HI of the FD device in FD communication systems to achieve higher capacity.

**Figure 5:**
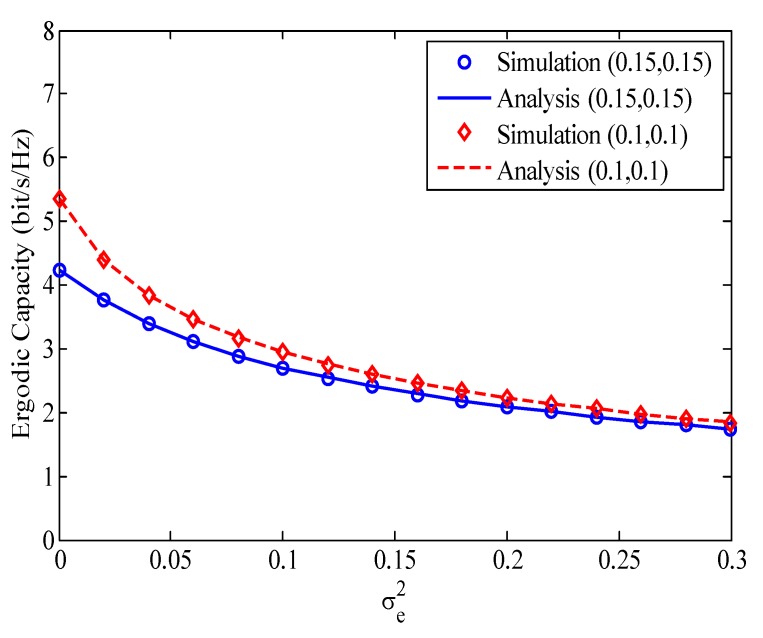
The effect of channel estimation error on the ergodic capacity of the considered HI-FD-MIMO relay system, SNR=30dB.

In Figure 5, we investigate the effect of the channel estimation error on the ergodic capacity of the considered HI-FD-MIMO relay system for two cases of the HI and RSI, i.e., k=l=0.15 and k=l=0.1. Compared with the case of P-CSI ($\sigma_{e}^{2}=0$), the ergodic capacity in the case of I-CSI is greatly reduced, even with small $\sigma_{e}^{2}$. On the other hand, when $\sigma_{e}^{2}$ varies from 0 to 0.15, the ergodic capacity rapidly reduces. However, when $\sigma_{e}^{2}$ increases from 0.15 to 0.3, the ergodic capacity slowly reduces. The combination of $\sigma_{e}^{2}$, HI, and RSI makes a strong degradation on the capacity of the considered system. We can see that in the case of P-CSI ($\sigma_{e}^{2}=0$), the capacities are 5.4 and 4.2 bit/s/Hz while they are 1.9 and 1.8 bit/s/Hz for $\sigma_{e}^{2}=0.3$ in the cases of (0.1,0.1) and (0.15,0.15), respectively.

**Figure 6:**
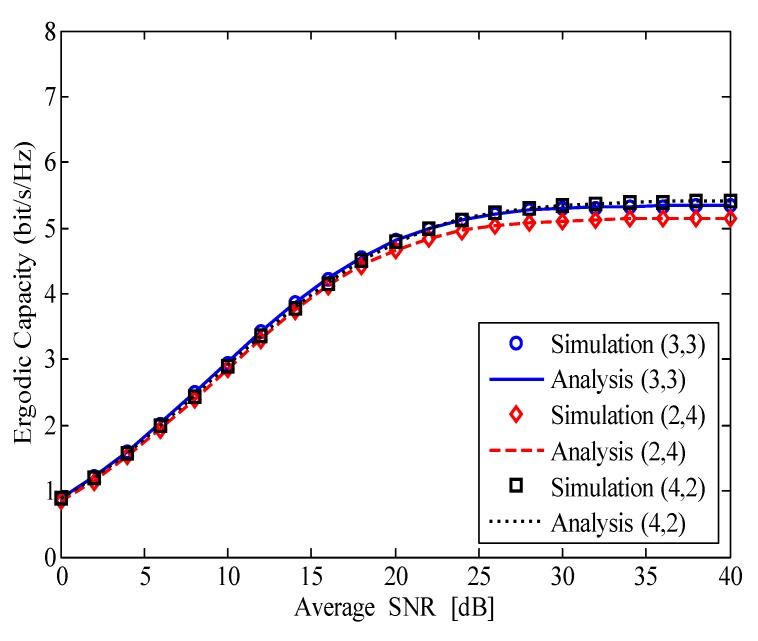
The ergodic capacity of the HI-FD-MIMO relay system with different numbers of reception and transmission antennas at the FD relay.

In Figure 6, we change the number of reception and transmission antennas of relay to consider its impact on the ergodic capacity of the considered HI-FD-MIMO relay system with $N_{r}+N_{t}=6$, $\sigma_{e}^{2}=0.001$, and k=l=0.1. It is obvious that the ergodic capacity in the case of (4,2) ($N_{r}=4;N_{t}=2$) is the best while the ergodic capacity in the case of (2,4) ($N_{r}=2;N_{t}=4$) is the worst. These results are reasonable for the considered system. It is because when the relay operates in FD mode, the usage of a larger number of transmission antennas will lead to stronger RSI power on the reception antennas of R. As given in (6) and (7), the SINDR at R but not at D is influenced by the RSI. Therefore, in the case of I-SIC, we need to use a larger number of reception antennas than the number of transmission antennas at the FD relay to improve the ergodic capacity of the considered HI-FD-MIMO relay system.

## 5. Conclusions

The full-duplex MIMO relay system is a candidate for future wireless networks due to the increasing capacity compared with traditional wireless systems. To consider the performance of a realistic FD-MIMO relay system, in this paper, we have conducted an investigation of the performance of the HI-FD-MIMO relay system with three imperfections, such as imperfect CSI, imperfect transceiver hardware, and imperfect SIC. We successfully obtained the exact closed-form expressions of outage probability and ergodic capacity of the considered HI-FD-MIMO relay system. Based on these expressions, the system performance is thoroughly determined under the impact of various parameters such as the channel estimation error, the HI level, the RSI level, and the number of reception and transmission antennas of FD relay. We observed that three imperfect factors caused the outage and capacity of the HI-FD-MIMO relay system to reach the saturation values faster. Thus, the wireless designers and researchers need to put more effort into reducing the channel estimation error, the HI, and the RSI levels before deploying the system in practical scenarios.
